# Early or First Aid Administration Versus Late or In-hospital Administration of Aspirin for Non-traumatic Adult Chest Pain: A Systematic Review

**DOI:** 10.7759/cureus.6862

**Published:** 2020-02-03

**Authors:** Therese Djarv, Janel M Swain, Wei-Tien Chang, David A Zideman, Eunice Singletary

**Affiliations:** 1 Emergency Medicine, Karolinska Institute, Stockholm, SWE; 2 Emergency Health Services, Nova Scotia, Dartmouth, CAN; 3 Emergency Medicine, National Taiwan University Hospital and College of Medicine, Taipei, TWN; 4 Pre-Hospital Emergency Medicine, Thames Valley Air Ambulance, Oxford, GBR; 5 Emergency Medicine, University of Virginia, Charlottesville, USA

**Keywords:** first aid, myocardial infarction, acetylic acid, asa

## Abstract

Chest pain is a common symptom of acute coronary syndrome, including myocardial infarction (MI). Treatment with antiplatelet agents, such as aspirin, improves survival, although the ideal dose is uncertain. It is unknown if outcomes can be improved by giving aspirin early in the course of MI as part of the first-aid management as opposed to late or in-hospital administration.

We searched the Medline, Embase, and Cochrane databases and used Grading of Recommendations, Assessment, Development, and Evaluation (GRADE) and Risk of Bias in Non-randomized Studies of Interventions (ROBINS-I) for determining the certainty of evidence. We included studies in adults with non-traumatic chest pain, where aspirin was administered early (within two hours) following the onset of chest pain as part of first-aid management as compared with late or in-hospital administration (The International Prospective Register of Systematic Reviews (PROSPERO) registration number: CDR153316). From 1470 references, we included three studies (one randomized controlled trial (RCT) and two non-RCTs). Early administration (median 1.6 hours or pre-hospital) was associated with increased survival as compared with late administration (median 3.5 hours or in-hospital) at seven days; risk ratio (RR) 1.04 (95% CI 1.03-1.06), 30 days RR 1.05 (95% 1.02-1.07), and one-year RR 1.06 (95% CI1.03-1.10). The evidence is of very low certainty due to limitations in study design and the imprecision of the evidence.

This systematic review would suggest that the early or first-aid administration of aspirin to adults with non-traumatic chest pain improves survival as compared with late or in-hospital administration.

## Introduction and background

Chest pain, which in adults is a common symptom of myocardial infarction (MI), results in more than eight million visits to emergency departments (EDs) each year in the US [1]. Between 13% and 24% of these visits for chest pain are due to acute coronary syndrome, which includes both ST-elevation myocardial infarction and non-ST-elevation acute coronary syndrome [2]. The administration of oral antiplatelet agents, such as aspirin, to individuals with non-traumatic chest pain has been shown to improve survival and is increasingly employed for chest pain in emergency care [3-4]. Given that aspirin is inexpensive and readily available, it is possible for first aid providers, such as family or friends, to initiate the administration of aspirin shortly after the onset of chest pain. However, since first aid providers would have to administer aspirin based on symptoms, it is important to elucidate the benefits and risks from the early or pre-hospital administration of aspirin prior to a confirmed diagnosis of an MI.

In 2015, the International Liaison Committee on Resuscitation (ILCOR) published a Consensus on Science and Treatment Recommendation (CoSTR), establishing the role of oral aspirin compared with placebo [5-6]. However, the optimal timing for aspirin administration is currently unknown. The ILCOR First Aid Task Force performed a systematic review to investigate the timing of aspirin administration for non-traumatic chest pain. This systematic review was designed to answer the question: Among adults with non-traumatic chest pain, does early or first aid administration of aspirin compared to late or in-hospital administration of aspirin change outcomes of survival, complications, incidence of cardiac arrest, cardiac functional outcome, infarction size, or resolution of chest pain?

## Review

Methods

ILCOR uses a continuous evidence process to evaluate evidence and develop treatment recommendations for resuscitation and relevant first aid topics, culminating in the publication of a CoSTR. This systematic review was conducted in accordance with the Cochrane Handbook for Systematic Reviews of Interventions, and reporting occurred through the Preferred Reporting Items for Systematic Reviews and Meta-Analyses (PRISMA) checklist [7]. The protocol for this systematic review was registered with The International Prospective Register of Systematic Reviews (PROSPERO, registration number: CRD153316).

Selection criteria

The following inclusion and exclusion criteria were used for the selection of articles:

Population

Included: Studies in 18 years or greater with non-traumatic chest pain. Studies of traumatic chest pain were excluded.

Intervention

Included: Studies where aspirin (oral, in any administration, any form, any dose), was administered early. Early administration was defined as administration in the pre-hospital or first aid phase or administration within two hours from onset of pain.

Excluded: Intravenous administration since it requires skills beyond typical first aid.

Comparison

Included: Studies where aspirin (oral, in any administration, any form, any dose) was administered late.

Late administration was defined as the administration of aspirin in hospital or administration after more than two hours from the onset of chest pain.

Outcomes

Outcomes were graded through consensus discussion by the ILCOR First Aid Task Force as “critical” or “important,” which ranged from 4-9 on a nine-point scale, according to the Grading of Recommendations, Assessment, Development, and Evaluation (GRADE) approach.

Primary outcomes: Survival (critical) at any reported time point (dichotomous outcome; yes/no). Complications (critical) - defined as any reported complication except for those classified as cardiac arrest (dichotomous outcome; yes/no). Incidence of cardiac arrest (critical) - defined as dysrhythmias associated with a cardiac arrest in a clinical situation, that is, ventricle fibrillation (VF), pulseless/sustained ventricle tachycardia (VT), asystole, pulseless electric activity, need for resuscitation (dichotomous outcome; yes/no).

Secondary outcomes: Cardiac functional outcome (important) - defined as any measurement of the function of the heart (continuous outcome). Infarct size (important) - defined as any measurement of an area (continuous outcome). Chest pain resolution (important) - defined as patients’ subjective opinion (dichotomous outcome; yes/no).

Study design

Included: Randomized controlled trials (RCTs) and non-randomized studies (non-randomized controlled trials, interrupted time series, controlled before-and-after studies, cohort studies) were eligible for inclusion.

The Task Force considered that it was possible that there would be limited evidence in the out-of-hospital setting. Therefore, studies performed in the in-hospital setting would be considered as indirect evidence if they were relevant to the PICO (Patient, Problem, or Population; Intervention; Comparison, control, or comparator; Outcome).

In addition, studies in which individuals had suspected or confirmed MI but could be generalized to the broader symptom of non-traumatic chest pain were included.

Excluded: Case series, unpublished studies, conference abstracts, and trial protocols were excluded.

Timeframe

All years and all languages were included as long as there was an English abstract available.

Search strategy and study selection

Search strings were developed by St. Michael’s Hospital library services for the following databases: Medline (OVID interface), Embase (OVID interface), and Cochrane. Databases were searched from their inception date until October 22, 2019. All search strategies can be found in Supplementary Materials 1.

Two authors (TD, JS) independently screened titles, abstracts, and full texts. Reasons for full-text exclusion, based on the selection criteria, were documented. Reference lists of all articles eligible for full-text screening were screened. Any discrepancies between authors were resolved by discussion with a third author (W-TC).

Data collection

One author extracted the following data: study design, study population, intervention, outcome measures (TD), and two authors independently assessed risk of bias (JS, W-TC). Discrepancies were discussed until consensus was reached between three authors (JS, W-TC, TD).

Risk of bias and certainty of evidence assessment

The GRADE approach was used to determine the certainty of evidence for the body of evidence [7]. GRADE assesses the risk of bias, indirectness, imprecision, inconsistency, and publication bias. The Risk of Bias in Non-randomized Studies of Interventions (ROBINS-I) was used for classifying the certainty of evidence in non-RCTs [7].

Data synthesis

Review Manager (RevMan 5.3, The Nordic Cochrane Centre, Copenhagen, Denmark, 2014) and GRADEPro software (Guideline Development Tool (Software), McMaster University, developed by Evidence Prime, Inc., available from gradepro.org) were used for data analysis and for creating forest plots. Dichotomous outcomes were reported as risk ratios (RR) with 95% confidence intervals (CIs). According to the PROSPERO registered protocol, we had planned to perform meta-analyses and subsequent subgroup analyses; however, an insufficient number of studies were identified. Forest plots were developed containing studies with different interventions that measured the same outcome. Heterogeneity was assessed by the visual inspection of the forest plot and by using the chi² test (significant if p <0.10) and the I² statistic (heterogeneity considered significant if I² >60%). Since we anticipated variation between studies, the random-effects model was used. A p-value of <0.05 was considered significant.

Results

Literature search and study selection

**Figure 1:**
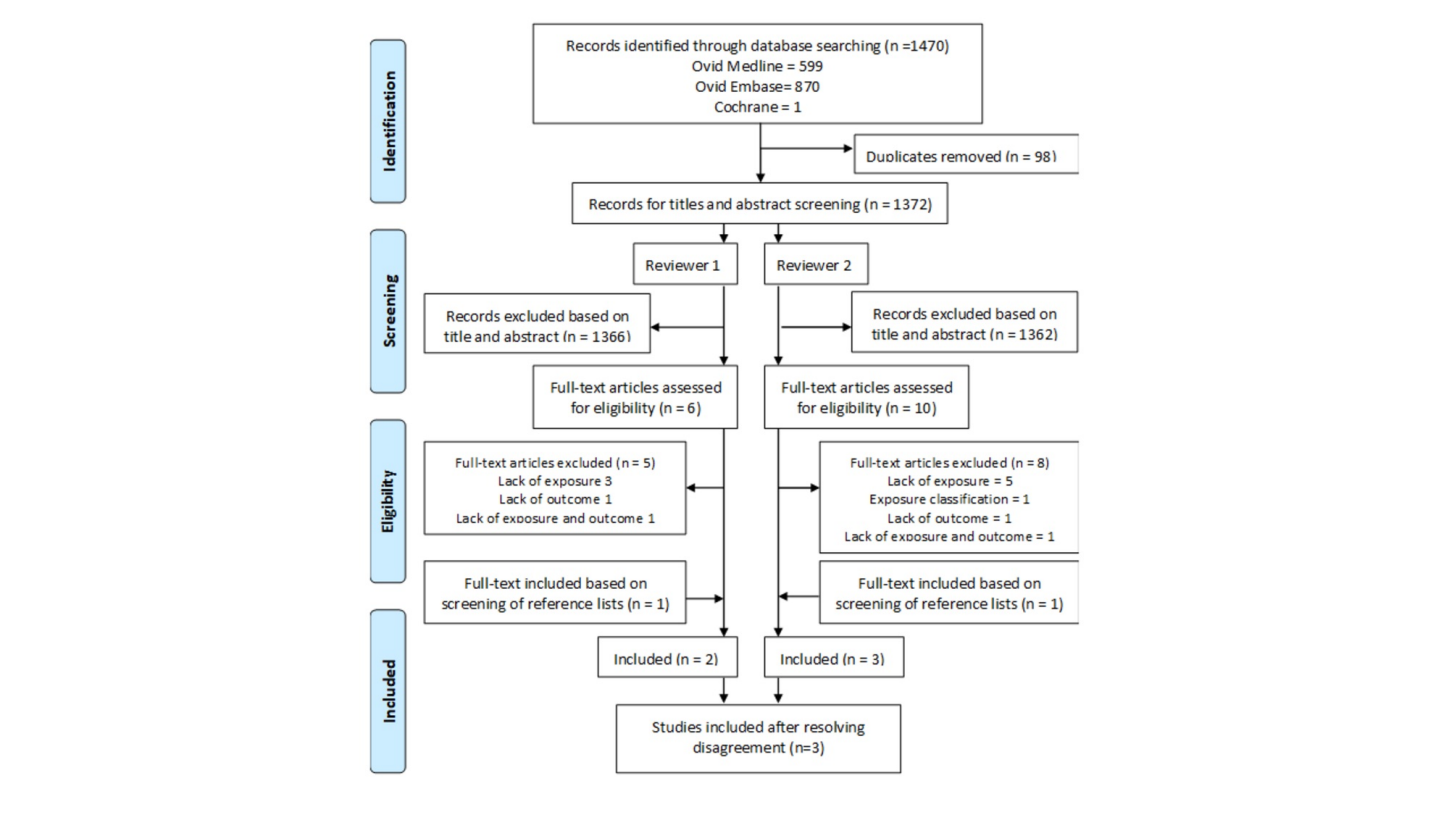
Study selection flow chart

We identified 1470 references through database searching. A Kappa value of 0.9 was calculated for inclusion following a full-text screening, indicating good inter-rater reliability. The main reason for excluding studies during the full-text screening was the lack of exposure (the declared time between the start of chest pain and the administration of aspirin). One study was added based on the reference list of one of the full-text screened studies, resulting in three studies being included [4,8-9]. Figure 1 illustrates the PRISMA study selection diagram, including reasons for article exclusion.

Study characteristics

We identified three studies from different geographical areas (Table 1), including one randomized controlled trial (RCT) and two non-RCTs [4,8-9].

**Table 1:** Study characteristics and findings CI: confidence interval

Author, year, country	Study design	Population	Intervention	Control	Findings
Second International Study of Infarct Survival Collaborative Group (ISIS-2), 1988, United Kingdom	Randomized controlled trial	17,187 suspected acute myocardial infarctions, of which 8587 got only aspirin (162.5 mg tablet daily for one month). A sub-group analysis of the aspirin arm was done.	Time from onset of pain to aspirin 0-2 hours (n=1309)	Time from the onset of pain to aspirin 2-24 hours (n= 7278)	Survival at 35 days relative risk 1.01 (95% CI 0.99-1.03)
Barbash, 2002, Israel	Non-randomized controlled trial (cohort)	922 consecutive acute myocardial infarction patients with ST-elevation in 26 hospitals. Cardiogenic shock was excluded. Clinical praxis was >200 mg aspirin.	Pre-hospital administration	Administration at hospital admission	Survival seven days relative risk 1.05 (95% 1.02-1.08). Survival at 30 days relative risk 1.07 (95% CI 1.03-1.11). Complications relative risk 0.56 (95% CI 0.44-0.71). Cardiac arrest relative risk 0.63 (95% CI 0.46-0.85).
Freimark, 2002, Israel	Non-randomized controlled trial (cohort) Posthoc-analysis of a randomized controlled trial on argatroban vs heparin as adjuvant treatment for thrombolysis in patients with acute myocardial infarction.	25 medical centers enrolled 1200 patients with acute myocardial infarction defined as >30 min chest pain & ST-elevation. Aspirin 160 mg should be given within 1 hr from the start of thrombolytic agents according to protocol and once daily for 30 days. Some patients received aspirin routinely before randomization incl. self-administration or personnel.	364 before thrombolytic agent. Median time to aspirin 1.6 hrs	836 after thrombolytic agent. Median time to aspirin: 3.5 hrs	Survival 7 days relative risk 1.04 (95% 1.01-1.06). Survival at 30 days relative risk 1.03 (95% CI 1.00-1.06). Complications relative risk 1.22 (95% CI 1.09-1.37). Cardiac arrest relative risk 1.53 (95% CI 1.12-2.08)

The ISIS-2 study compared time to aspirin (oral 160 mg) within one of four arms in an RCT of acute suspected MI; the time to aspirin administration was classified following consensus discussion by the ILCOR First Aid Task to early (0-2 hours from pain onset or pre-hospital to administration) compared with late administration (more than two hours from pain onset or in-hospital administration) [4]. Freimark et al. performed a post-hoc analysis of aspirin administration (oral 160 mg) before (early) or after (late, in-hospital) giving a thrombolytic agent to individuals with acute MI with ST-segment elevation [9].

Barbash et al. compared pre-hospital (early) with in-hospital (late) administration of aspirin (>200 mg oral) in a prospective national survey of AMI patients with ST-segment elevation and Killip class I-III (a classification of heart failure) on admission [8].

For the important outcomes of cardiac functional outcome and infarct size, as well as for the secondary important outcome of chest pain resolution, there were no comparator studies evaluating the time of aspirin administration.

Study findings

Survival

**Figure 2:**
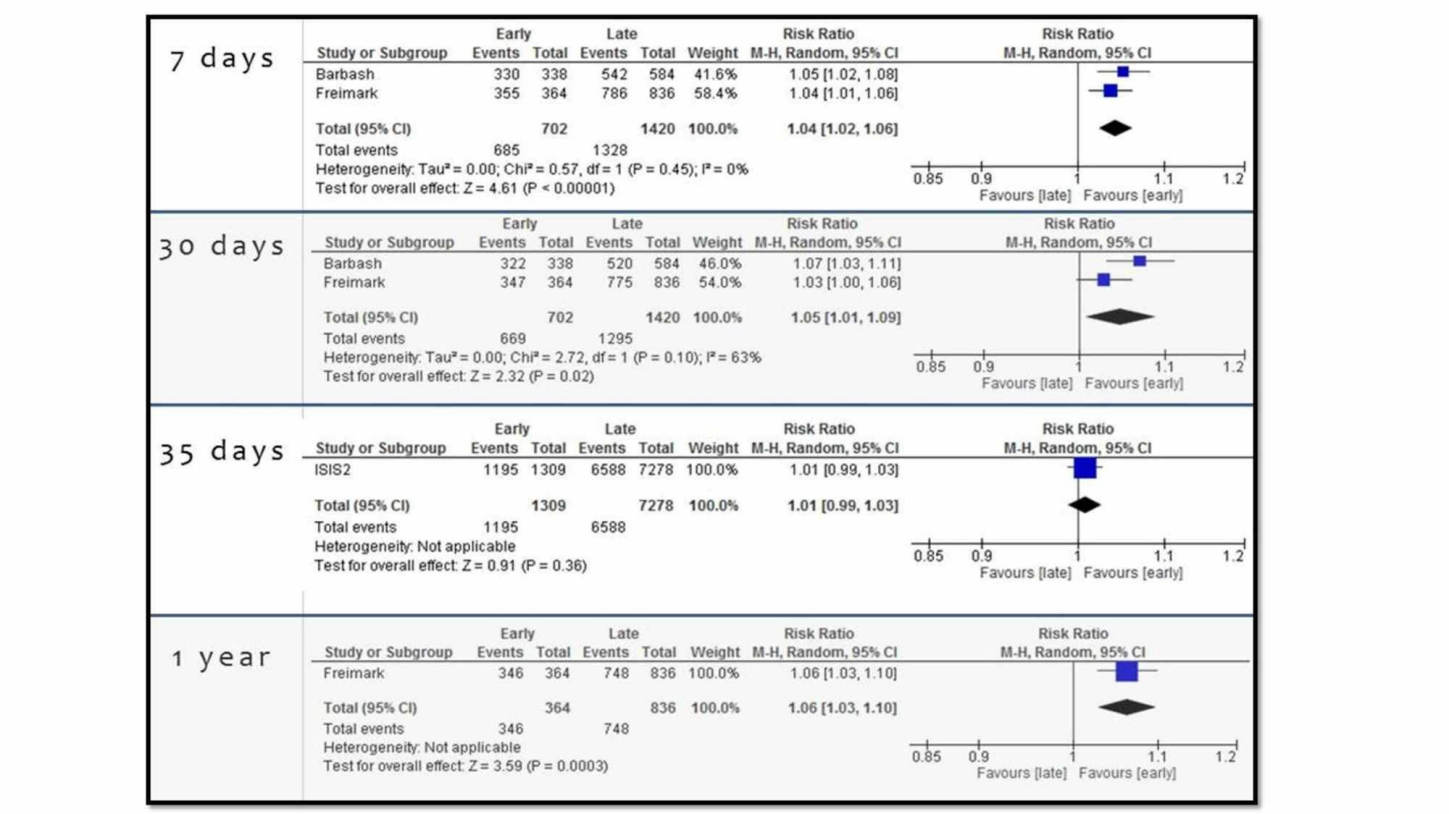
Forest plots for the critical outcome survival at different time points. M-H: Mantel-Haenszel

Freimark et al. and Barbash et al. evaluated the critical outcome survival at seven days in 2122 subjects with an ST-elevation acute MI [8-9]. A benefit was found for the early administration of aspirin (median 1.6 hours from pain onset to 160 mg aspirin administration and >200 mg aspirin administered pre-hospitally, respectively) compared with late or in-hospital administration (97.5% compared with 93.5% (p<0.001, RR, 1.04; 95% CI, 1.02-1.06)); 37 more subjects per 1000 treated survived to seven days with the early administration of aspirin (95% CI, from 18 more to 56 more) (Figure 2).

The same studies also evaluated the critical outcome of survival at 30 days, again showing a benefit for the early administration of aspirin as compared to late administration (95.2% versus 91.2% (RR, 1.05; 95% CI, 1.01-1.09)); 46 more subjects per 1000 treated survived to 30 days with the early administration of aspirin (95% CI from nine more to 82 more). There was non-important to moderate heterogeneity between these subgroups.

For the critical outcome of survival at 35 days, the Second International Study of Infarct Survival (ISIS-2) study enrolling 8587 subjects with acute MI found no benefit from aspirin administration (162.5 mg, enteric-coated) within two hours of the onset of symptoms compared with late administration (91.2% versus 90.5% (RR, 1.01; 95% CI, 0.99-1.03)); nine more subjects per 1000 treated survived to 35 days with the early administration of aspirin (95% CI from nine fewer to 27 more) [4].

Finally, Freimark et al.'s study enrolling 1200 subjects with acute ST-elevation MI evaluated the critical outcome of survival at one year, with findings showing benefit from early aspirin administration (median 1.6 hours from pain onset receiving 160 mg aspirin) compared with late administration (95.0% versus 89.4% (RR, 1.06; 95% CI, 1.03-1.10)); 54 more patients per 1000 treated survived to one year with early administration of aspirin (95% CI from 26 more to 89 more) [9].

Complications

**Figure 3:**
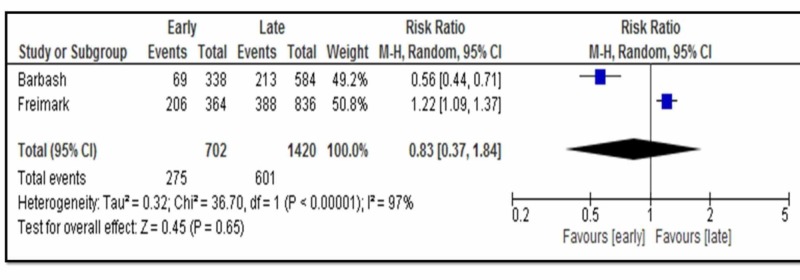
Forest plot for critical outcome complications M-H: Mantel-Haenszel

Two observational studies, i.e. Freimark et al. and Barbash et al., evaluated the critical outcome of complications, together including a total of 2122 subjects with acute ST-elevation MI, finding no significant difference in the risk of developing complications with early administration (median 1.6 hours from pain onset receiving 160 mg aspirin and >200 mg aspirin pre-hospitally, respectively) as compared with late administration (median 3.5 hours from pain onset receiving 160 mg aspirin and >200 mg aspirin in hospital) (RR, 0.83; 95% 0.37-1.84) [8-9]. There was considerable heterogeneity between the subgroups (Figure 3).

Incidence of cardiac arrest

No studies reported the outcome of cardiac arrest; however, two studies reported conflicting results regarding complications that the ILCOR First Aid Task Force, in consensus, interpreted as a likely cardiac arrest.

Barbash et al. reported dysrhythmias associated with cardiac arrest (asystole, sustained ventricular tachycardia (VT), and primary ventricular fibrillation (VF)) in a total of 922 subjects with acute ST-elevation MI but found no significant difference between those with early administration (>200 mg aspirin administered pre-hospitally) as compared with late administration (>200 mg aspirin administered at hospital arrival) (RR 0.82, 95% 0.56-1.20) [8]. The same study also showed a reduced risk of the need for resuscitation among those who received early compared with the late administration of aspirin (RR 0.38, 95%CI 0.20-0.69).

**Figure 4:**
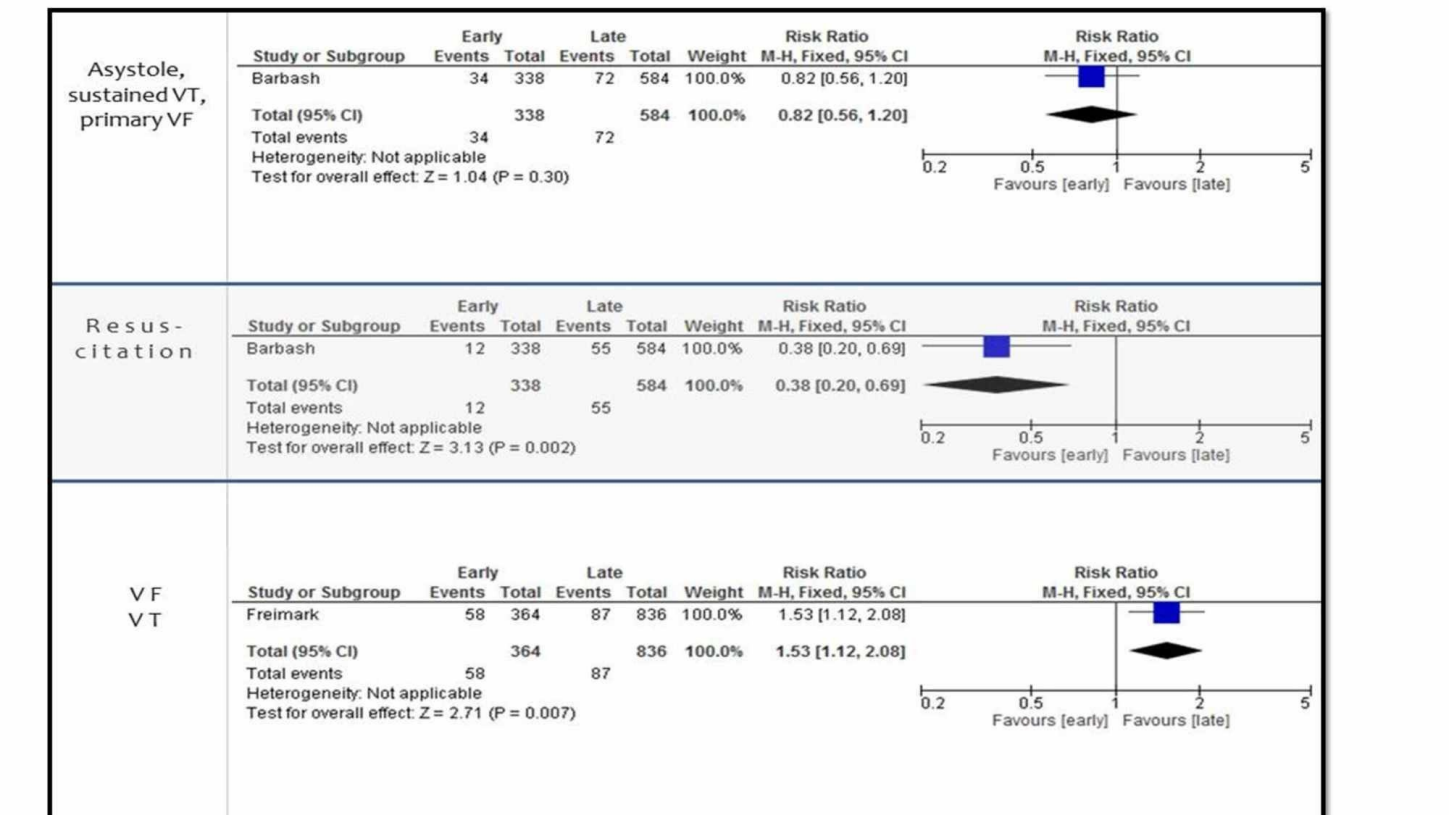
Forest plots for the critical outcome incidence of cardiac arrest M-H: Mantel-Haenszel; VT: Ventricle tachycardia; VF: Ventricle fibrillation

Freimark et al. reported a higher incidence of dysrhythmias associated with cardiac arrest (VF/VT) in a study enrolling 1200 subjects with acute ST-elevation MI in the group that received early aspirin (median 1.6 hours from pain onset to 160 mg aspirin administration) compared with late aspirin (median 3.5 hours from pain onset to 160 mg aspirin administration) (RR 1.53; 95% CI, 1.12-2.08) (Figure 4) [9].

Risk of bias and certainty of the evidence

The confidence in the estimate of effect for the outcomes evaluated was lowered for a number of reasons. For the outcome survival at 35 days in the GRADE EP tables, the certainty of estimate was decreased because of serious indirectness as a result of the difference in the studied population (MI) as compared with the target population (non-traumatic chest pain). In both cohort studies, there was a risk of bias in the form of confounding by indications due to only including subjects with typical symptoms [8-9]. In one study, there was a risk of bias due to missing data and misclassification since an exact time was reported for aspirin administration in only 104/364 in the early group as compared with 809/836 in the late group [8]. An insufficient number of studies were included to generate funnel plots to judge the publication bias. Table 2 includes GRADE tables.

**Table 2:** GRADE tables RR: relative risk a. Randomized trial of intravenous streptokinase, oral aspirin, both, or neither among 17,187 cases of suspected acute myocardial infarction: ISIS-2. ISIS-2 (Second International Study of Infarct Survival) Collaborative Group. 1988; b. Looked at myocardial infarction patients only, not just chest pain; c. Barbash I et al., 2002, Freimark D et al., 2002; d. There was no control for confounding variables (including thrombolysis and not controlling for underlying disease/health); e. Freimark D et al., 2002; f. Barbash I et al., 2002

Certainty assessment							№ of patients		Effect		Certainty	Importance
№ of studies	Study design	Risk of bias	Inconsistency	Indirectness	Imprecision	Other considerations	Aspirin be administered early	Late	Relative (95% CI)	Absolute (95% CI)		
35 days survival												
1 ^a^	Randomized trials	Not serious	Not serious	Very serious ^b^	Not serious	None	1195/1309 (91.3%)	6588/7278 (90.5%)	RR 1.01 (0.99 to 1.03)	9 more per 1 000 (from 9 fewer to 27 more)	⨁⨁◯◯ LOW	CRITICAL
7 days survival												
2 ^c^	Observational studies	Serious ^d^	Not serious	Serious ^b^	Not serious	None	685/702 (97.6%)	1328/1420 (93.5%)	RR 1.04 (1.02 to 1.06)	37 more per 1 000 (from 19 more to 56 more)	⨁⨁◯◯ LOW	CRITICAL
30 days survival												
2 ^c^	Observational studies	Serious ^d^	Not serious	Serious ^b^	Not serious	None	669/702 (95.3%)	1295/1420 (91.2%)	RR 1.05 (1.01 to 1.09)	46 more per 1 000 (from 9 more to 82 more)	⨁◯◯◯ VERY LOW	CRITICAL
1 year survival												
1 ^e^	Observational studies	Serious	Not serious	Serious ^b^	Not serious	None	346/364 (95.1%)	748/836 (89.5%)	RR 1.06 (1.03 to 1.10)	54 more per 1 000 (from 27 more to 89 more)	⨁⨁◯◯ LOW	CRITICAL
Complications												
2 ^c^	Observational studies	Serious	Not serious	Serious ^b^	Not serious	None	275/702 (39.2%)	601/1420 (42.3%)	RR 0.83 (0.37 to 1.84)	72 fewer per 1 000 (from 267 fewer to 356 more)	⨁⨁◯◯ LOW	CRITICAL
Cardiac arrest Freimark												
1 ^e^	Observational studies	Serious ^b^	Not serious	Serious ^b^	Not serious	None	58/364 (15.9%)	87/836 (10.4%)	RR 1.53 (1.12 to 2.08)	55 more per 1 000 (from 12 more to 112 more)	⨁⨁◯◯ LOW	CRITICAL
Cardiac arrest Barbash												
1 ^f^	Observational studies	Serious ^b^	Not serious	Serious ^b^	Not serious	None	34/338 (10.1%)	72/584 (12.3%)	RR 0.82 (0.56 to 1.20)	22 fewer per 1 000 (from 54 fewer to 25 more)	⨁⨁◯◯ LOW	CRITICAL

Discussion

This systematic review searched for the benefits associated with the early or first aid administration as compared with late or in-hospital administration of aspirin to adults with non-traumatic chest pain. The hypothesis behind comparing early with late administration is that the time to treatment with simple antiplatelet agents, such as aspirin, a drug readily available in the first aid or pre-hospital environment, may improve survival in patients with a possible MI [3].

Two non-RCTs were identified, which support early administration, including self-administration, for the outcome of improved survival at seven and 30 days while one RCT showed no significant difference in survival between early and late aspirin administration at 35 days. We were not able to perform a meta-analysis, including all three studies, due to heterogeneous populations (suspected MI versus ST-segment elevation acute MI), different study designs (cohort versus RCT), and the studies were performed at different chronological times (1988 versus 2002). The latter was considered important by the Task Force as clinical practice, for example, reperfusion therapy, had changed in the 14-year study gap.

Conflicting results were found for the critical outcomes of the complications and incidence of cardiac arrest. In one study, the cohort receiving early aspirin suffered more in-hospital arrhythmias, however, this complication did not affect the overall survival of this cohort [9]. Reasons for conflicting results regarding arrhythmias and survival might relate to the time point of the ventricular arrhythmias since those occurring early in the time course of the clinical event come with a higher incidence of survival than with late arrhythmias [10-11].

These results have important implications for individuals with symptoms of non-traumatic chest pain, their family members, and first aid providers. Based on the current evidence, the early or first aid administration of aspirin has a beneficial effect on short- and long-term survival. However, the exact dose of administered aspirin and the critical time window of this early administration remains unknown. Further, in individuals with other etiologies of non-traumatic chest pain, such as gastrointestinal or large vessel disease, there might be a concern of the potential risk of bleeding from aspirin administration.

Currently, there are a lack of studies on aspirin administration by first aid providers and its associated benefits and risks. One previous study by Quan et al. found no adverse effects after the administration of aspirin by pre-hospital personnel to adults with presumed acute coronary syndromes [12]. From a risk perspective, it is unknown if there is an increased risk of bleeding from the administration of a single dose of aspirin. Bleeding within the group of patients with an MI has increased during the last two decades and is now around 2%, with the increase mainly attributed to the use of invasive treatment strategies known to affect the risk of bleeding, yet with the benefit of improved survival [13].

Limitations

Our review has several limitations. The ISIS-2 study was completed 30 years ago, and even if the population and exposure are comparable to today’s care, the outcome of survival after an MI is better today [4]. Further, ISIS-2 was performed before the routine use of thienopyridine for acute coronary syndrome. It is possible that the benefit of early acetylsalicylic acid (ASA) diminished with rapid thienopyridine treatment, which is standard for PCI patients nowadays. The other two included studies are from the same authors, country, and year, which might limit generalizability. Further, both Barbash et al. and Freimark et al. included acute MI with ST-elevation rather than the wider target population with non-traumatic chest pain, and there were no studies using undifferentiated chest pain as the target population [8-9]. Also, one study assessed aspirin administration before as compared to aspirin administration after a thrombolytic agent, which might affect aspirin’s effect [9]. The exact time to aspirin administration was only available in one-third of the early group, which limits the validity of the median time to aspirin administration (1.6 hrs). In summary, the evidence in the included studies is of very low certainty because of the risk of bias, imprecision, and indirectness in the studies.

## Conclusions

The early oral aspirin administration, including self-administration, appears to have a raised short- and long-term survival ratio as compared to the late administration of aspirin in subjects with non-traumatic chest pain typical of an acute MI. Future research should focus on the early administration of aspirin in the wider population with non-traumatic chest pain, the exact dose of aspirin to be administered, and the critical time window from onset of pain to administration of aspirin.

## Appendices

Search string OVID/PubMed

((((((("Chest Pain"[Mesh] OR "Chest Pain"[All Fields]OR "Angina Pectoris"[Mesh] OR “Myocardial Infarction/drug therapy*"[Mesh] OR “Myocardial Infarction/therapy*"[Mesh] OR “Chest Pain/drug therapy*” [Mesh] OR angina[TIAB] ))) AND (("Aspirin"[Mesh] OR “Aspirin/therapeutic use*”[Mesh] OR “Aspirin/administration & dosage*"[Mesh] OR "acetylsalicylic acid"[TIAB] OR "Aspirin"[All Fields]))) AND (("Emergency Medical Services*"[Mesh] OR "Emergency Service, Hospital"[Mesh] OR "Emergency Treatment"[Mesh] OR "Emergencies"[Mesh] OR “prehospital”[TIAB] OR “pre-hospital”[TIAB] OR “ems”[All Fields] OR “out-of-hospital”[All Fields] OR “early”[All Fields] OR “earlier”[All Fields])))) AND ((((((((("randomized controlled trial"[PT] OR "controlled clinical trial"[PT] OR "clinical trial"[PT] OR "comparative study"[PT] OR random*[TIAB] OR controll*[TIAB] OR "intervention study"[TIAB] OR "experimental study"[TIAB] OR "comparative study"[TIAB] OR trial[TIAB] OR evaluat*[TIAB] OR "Before and after"[TIAB] OR "interrupted time series"[TIAB]))) OR (("Epidemiologic Studies"[Mesh] OR "case control"[TIAB] OR "case-control"[TIAB] OR ((case[TIAB] OR cases[TIAB]) AND (control[TIAB] OR controls[TIAB)) OR "cohort study"[TIAB] OR "cohort analysis"[TIAB] OR "follow up study"[TIAB] OR "follow-up study"[TIAB] OR "observational study"[TIAB] OR "longitudinal"[TIAB] OR "retrospective"[TIAB] OR "cross sectional"[TIAB] OR "cross-sectional"[TIAB] OR questionnaire[TIAB] OR questionnaires[TIAB] OR survey[TIAB])))) NOT (("animals"[MH] NOT (“animals”[MH] AND "humans"[MH])))) NOT (("letter"[pt] OR "comment"[pt] OR "editorial"[pt] OR “conference"[pt] OR “review” [pt])))) AND English[lang]))

Search string Embase

((((((("Chest Pain" OR "Chest Pain" OR "Angina Pectoris" OR “angina” OR “Myocardial Infarction/drug therapy*" OR “Myocardial Infarction/therapy*" ))) AND (("Aspirin" OR “Aspirin/therapeutic use*” OR "acetylsalicylic acid" OR "aspirin"))) AND (("Emergency Medical Services" OR "Emergency Service, Hospital" OR "Emergency Treatment" OR "Emergencies" OR “Prehospital” OR “Pre-hospital” OR “ems” OR “out-of-hospital” OR early OR earlier)))) AND ((((((((("randomized controlled trial" OR "controlled clinical trial" OR "clinical trial" OR "comparative study" OR random* OR controll* OR "intervention study" OR "experimental study" OR "comparative study" OR trial OR evaluat* OR "Before and after" OR "interrupted time series"))) OR (("Epidemiologic Studies" OR "case control" OR "case-control" OR ((case OR cases) AND (control OR controls)) OR "cohort study" OR "cohort analysis" OR "follow up study" OR "follow-up study" OR "observational study" OR "longitudinal" OR "retrospective” OR "cross sectional" OR "cross-sectional" OR questionnaire OR questionnaires OR survey)))) NOT (("animals" NOT (animals AND "humans")))) NOT (("letter" OR "comment" OR "editorial" OR “conference” OR “review”)))) AND English))
